# Inactivation of the Monofunctional Peptidoglycan Glycosyltransferase SgtB Allows *Staphylococcus aureus* To Survive in the Absence of Lipoteichoic Acid

**DOI:** 10.1128/JB.00574-18

**Published:** 2018-12-07

**Authors:** Eleni Karinou, Christopher F. Schuster, Manuel Pazos, Waldemar Vollmer, Angelika Gründling

**Affiliations:** a Section of Microbiology and MRC Centre for Molecular Bacteriology and Infection, Imperial College London, London, United Kingdom; b The Centre for Bacterial Cell Biology, Newcastle University, Newcastle upon Tyne, United Kingdom; Princeton University

**Keywords:** *Staphylococcus aureus*, cell wall, lipoteichoic acid

## Abstract

The cell wall of Staphylococcus aureus is composed of peptidoglycan and the anionic polymers lipoteichoic acid (LTA) and wall teichoic acid. LTA is required for growth and normal cell morphology in S. aureus. Strains lacking LTA are usually viable only when grown under osmotically stabilizing conditions or after the acquisition of compensatory mutations. LTA-negative suppressor strains with inactivating mutations in *gdpP*, which resulted in increased intracellular c-di-AMP levels, were described previously. Here, we sought to identify factors other than c-di-AMP that allow S. aureus to survive without LTA. LTA-negative strains able to grow in unsupplemented medium were obtained and found to contain mutations in *sgtB*, *mazE*, *clpX*, or *vraT*. The growth improvement through mutations in *mazE* and *sgtB* was confirmed by complementation analysis. We also showed that an S. aureus *sgtB* transposon mutant, with the monofunctional peptidoglycan glycosyltransferase SgtB inactivated, displayed a 4-fold increase in the MIC of oxacillin, suggesting that alterations in the peptidoglycan structure could help bacteria compensate for the lack of LTA. Muropeptide analysis of peptidoglycans isolated from a wild-type strain and *sgtB* mutant strain did not reveal any sizable alterations in the peptidoglycan structure. In contrast, the peptidoglycan isolated from an LTA-negative *ltaS* mutant strain showed a significant reduction in the fraction of highly cross-linked peptidoglycan, which was partially rescued in the *sgtB ltaS* double mutant suppressor strain. Taken together, these data point toward an important function of LTA in cell wall integrity through its necessity for proper peptidoglycan assembly.

**IMPORTANCE** The bacterial cell wall acts as a primary defense against environmental insults such as changes in osmolarity. It is also a vulnerable structure, as defects in its synthesis can lead to growth arrest or cell death. The important human pathogen Staphylococcus aureus has a typical Gram-positive cell wall, which consists of peptidoglycan and the anionic polymers LTA and wall teichoic acid. Several clinically relevant antibiotics inhibit the synthesis of peptidoglycan; therefore, it and teichoic acids are considered attractive targets for the development of new antimicrobials. We show that LTA is required for efficient peptidoglycan cross-linking in S. aureus and inactivation of a peptidoglycan glycosyltransferase can partially rescue this defect, together revealing an intimate link between peptidoglycan and LTA synthesis.

## Abstract (precis)

The bacterial cell wall acts as a primary defense against environmental insults such as changes in osmolarity. It is also a vulnerable structure, as defects in its synthesis can lead to growth arrest or cell death. The important human pathogen Staphylococcus aureus has a typical Gram-positive cell wall, which consists of peptidoglycan and the anionic polymers LTA and wall teichoic acid. Several clinically relevant antibiotics inhibit the synthesis of peptidoglycan; therefore, it and teichoic acids are considered attractive targets for the development of new antimicrobials. We show that LTA is required for efficient peptidoglycan cross-linking in S. aureus and inactivation of a peptidoglycan glycosyltransferase can partially rescue this defect, together revealing an intimate link between peptidoglycan and LTA synthesis.

## INTRODUCTION

Staphylococcus aureus is a Gram-positive bacterium found as a commensal on the skin and in nasal passages of healthy individuals. However, this bacterium is also an important human pathogen causing hospital-acquired and community-acquired infections, such as serious skin infections, osteomyelitis, and endocarditis (1–3). Of major concern is the increasing resistance of this organism to a large number of clinically relevant antibiotics (4). A number of virulence factors contribute to successful host colonization, immune evasion, and acquisition of nutrients within the host (5). Many of these factors are either secreted proteins or other extracellular proteins closely associated with the bacterial cell envelope (6).

The cell envelope is essential for bacterial survival and pathogenesis but also is a target of a number of important antimicrobials. It functions as a barrier and protects bacteria from environmental insults but at the same time needs to allow the passage of solutes and nutrients, as well as sensing of changes in the external environment (7). S. aureus has a typical Gram-positive cell envelope, which consists of a cytoplasmic membrane surrounded by a thick peptidoglycan layer (8). The peptidoglycan layer is a dynamic macromolecular structure that undergoes constant cycles of polymerization and hydrolysis to allow bacteria to grow and to divide (7). It is composed of glycan chains made of alternating *N*-acetylglucosamine and *N*-acetylmuramic acid residues connected by peptide bridges (9). This mesh-like sacculus is able to protect the cell from environmental threats while withstanding the high internal osmotic pressure (10). The final steps of peptidoglycan synthesis are catalyzed by enzymes termed penicillin-binding proteins (PBPs), and coordinated actions of these enzymes are crucial for cell survival (11). PBPs with glycosyltransferase and transpeptidase activities polymerize the glycan chains and form peptide cross-bridges, while monofunctional transpeptidases have only the former activity (11).

S. aureus encodes four PBPs, of which PBP1, which has transpeptidase activity, and the bifunctional PBP2, which has transpeptidase and glycosyltransferase activities, are the minimal requirements for cell survival (12). In methicillin-resistant S. aureus (MRSA) strains, the alternative PBP2A, which has transpeptidase activity, is needed for β-lactam resistance, in addition to the glycosyltransferase activity of PBP2 (13, 14). Moreover, additional nonessential proteins that are involved in peptidoglycan synthesis, such as the monofunctional glycosyltransferases SgtA and SgtB (also named Mgt), have been identified (12). Previous studies have shown that, although SgtA and SgtB have glycosyltransferase activity *in vitro*, only SgtB can support the growth of S. aureus in the absence of the main glycosyltransferase PBP2. SgtB alone, however, cannot support the growth of *S. aureus* in the presence of β-lactam antibiotics when an interaction between PBP2 and PBP2A is needed (15, 16).

In Gram-positive bacteria, the peptidoglycan layer is interspersed with a plethora of proteins and cell wall polymers named teichoic acids (17). Teichoic acids are further categorized into lipoteichoic acid (LTA), which is anchored to the outer leaflet of the cytoplasmic membrane via a lipid moiety, and wall teichoic acid (WTA), which is covalently attached to the peptidoglycan (18). Teichoic acids form an important part of the cell wall and contribute to the physical and chemical properties of the cell wall and to the binding of divalent cations (19, 20). While both WTA and LTA are polyanionic cell wall polymers, they are synthesized through separate independent pathways in S. aureus and many other Gram-positive bacteria (21, 22). Consistent with this, our work on the Gram-positive pathogen Listeria monocytogenes has revealed that LTA synthesis is not abrogated in the absence of WTA and vice versa (23). Recent work using pathway-specific inhibitors and a gene interaction screen provided further evidence that the polymers not only are synthesized through separate pathways but also have distinct functions in S. aureus (24).

LTA is an anionic polymer that in S. aureus is composed of glycerolphosphate repeating units, which are further decorated with d-alanine residues and, under high salt conditions, also with *N*-acetylglucosamine residues, as shown recently (25, 26). Most proteins required for LTA synthesis have been identified and extensively studied over the years (22, 27). One of the key enzymes required for LTA synthesis is the LTA synthase enzyme LtaS (28, 29); this enzyme polymerizes the LTA backbone chain on the outside of the cell, using the glycerolphosphate head group of the membrane lipid phosphatidylglycerol as the substrate (29, 30). LTA is indispensable for the growth of S. aureus under standard laboratory growth conditions, which highlights its important physiological role (29, 31).

Previous studies indicated functions for LTA in helping to direct the cell division machinery (32), in controlling autolysin activity (31), in facilitating biofilm formation (33), in mediating interactions with host cell receptors (17), in controlling susceptibility and/or resistance to antimicrobial peptides, and in maintaining cation homeostasis (20). S. aureus *ltaS* mutants, which lack the complete LTA polymer, can be constructed in some strain backgrounds at low growth temperatures (31). It has also been shown that LTA-deficient S. aureus strains are viable when grown under osmotically stabilizing conditions in medium containing 7.5% NaCl or 40% sucrose (31, 34). However, the bacteria display severe morphological defects, including increased cell size, clustering, and cell division defects, even under conditions permissive for growth (34). Bacteria can also readily acquire compensatory mutations, allowing them to grow in unsupplemented medium and improving their morphological defects (34). The majority of compensatory mutations previously observed were in *gdpP*, leading to inactivation of the c-di-AMP phosphodiesterase GdpP (34). The resulting increase in cellular c-di-AMP levels allowed the bacteria to survive the cell wall stress caused by the absence of LTA, which is now thought to be due to changes in the osmotic balance in the cell (34). However, compensatory mutations that rescued the growth of an LTA-negative S. aureus strain were found not only in *gdpP* but also in other genes (34).

As part of the current study, we sought to identify these genes and to further characterize the encoded proteins, to gain additional insight into why LTA is essential for the growth of S. aureus and potentially to uncover novel cellular functions for proteins involved in cell wall assembly or maintenance. Using a suppressor screen approach, we found that inactivation of the monofunctional peptidoglycan glycosyltransferase SgtB allowed S. aureus to grow in the absence of LTA. We further showed that peptidoglycan cross-linking was significantly reduced in the absence of LTA and could be partially restored upon inactivation of SgtB. This might strengthen the peptidoglycan layer and thus contribute to the observed growth rescue.

## RESULTS

### Identification of *S. aureus* suppressor strains able to grow in the absence of LTA in a c-di-AMP-independent way.

**FIG 1:**
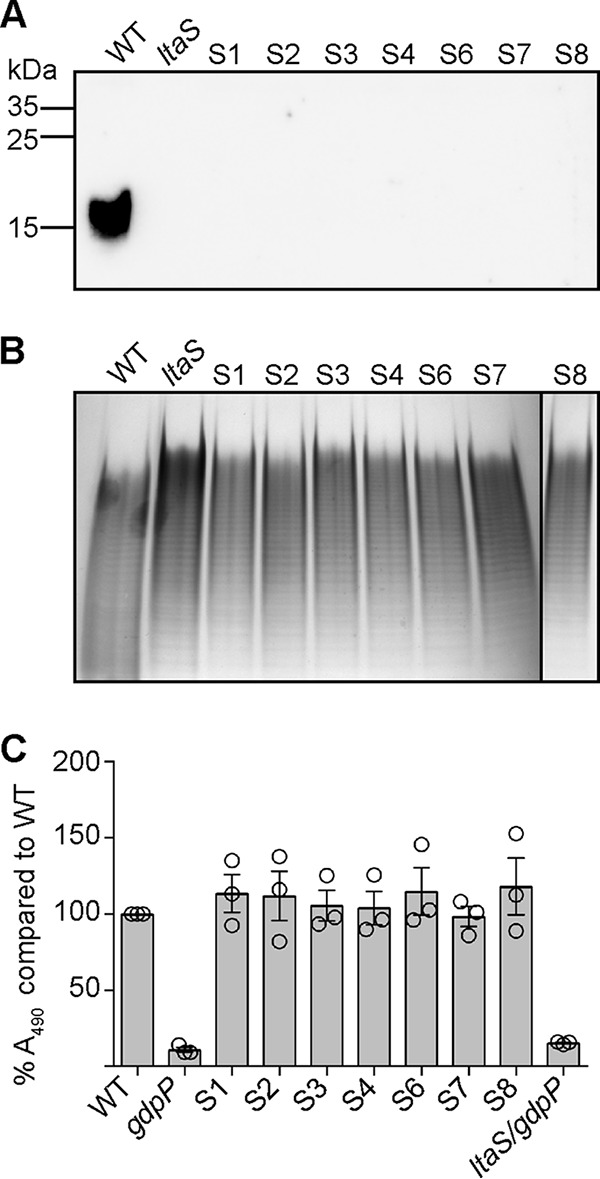
Detection of LTA by Western blotting, WTA by Alcian blue/silver staining, and c-di-AMP by using a competitive ELISA method. (A) Detection of LTA by Western blotting. Cell extracts were prepared from LAC* (WT strain), an *ltaS* mutant strain (strain ANG2135), and the 7 *ltaS* suppressor strains (strains S1 to S4 and S6 to S8) and separated on a 15% polyacrylamide gel. LTA was subsequently detected by Western blotting using a polyglycerolphosphate-specific monoclonal antibody. A representative result from three independent experiments is shown. (B) Visualization of WTA by native PAGE. WTAs were isolated from the same strains as shown in panel A and separated on a 20% native gel. Bands were visualized with Alcian blue staining and subsequently silver staining. One representative result from four independent experiments is shown. (C) c-di-AMP detection by competitive ELISA. Cytoplasmic extracts were prepared from LAC* (WT strain), the high-c-di-AMP-level control strains LAC* *gdpP*::*kan* (*gdpP*) and US3 (*ltaS gdpP*), and the 7 *ltaS* suppressor strains (strains S1 to S4 and S6 to S8). c-di-AMP amounts were determined and compared to those of the WT strain by ELISA. Of note, because this was a competitive ELISA, lower *A*_490_ readings were obtained for samples with higher c-di-AMP levels. The *A*_490_ reading obtained for the sample derived from the WT strain was set to 100%, and percentage values were calculated for the test strains. The average percentage values and SDs from three independent experiments (with three technical replicates) are plotted.

LTA-deficient S. aureus strains are viable when grown under osmotically stabilizing conditions in medium containing 7.5% NaCl or 40% sucrose or in unsupplemented medium after the acquisition of compensatory mutations (31, 34). The majority of compensatory mutations observed previously were in *gdpP*, leading to inactivation of the c-di-AMP phosphodiesterase GdpP (34). In the same study, suppressor strains with mutations outside the *gdpP* gene were noted (34). In order to characterize suppressor strains with mutations in genes other than *gdpP* and to gain further insight into the cellular function of LTA, a larger suppressor screen was performed and the *ltaS* mutant LAC*Δ*ltaS*::*erm* strains constructed in medium supplemented with either sucrose or NaCl were plated on unsupplemented tryptic soy agar (TSA) plates. A number of independently obtained suppressor colonies were subsequently passed four times in fresh tryptic soy broth (TSB) to further improve their growth. Next, the chromosomal DNA was isolated from 80 suppressor strains, and those lacking mutations in *gdpP* (coding for the c-di-AMP hydrolase) and *dacA* (coding for the c-di-AMP cyclase enzyme) were identified by determining the sequences of these two genes. Of 80 colonies screened, no strains had mutations in *dacA* and 17 strains had no mutation in *gdpP*, 7 of which were selected for further analysis. The absence of LTA and the presence of WTA in the suppressor strains were confirmed by Western blotting and Alcian blue/silver staining analysis, respectively (Fig. 1A and B). Next, the relative cellular c-di-AMP levels in the different strains were determined using a previously described competitive enzyme-linked immunosorbent assay (ELISA) (35, 36) (Fig. 1C). In contrast to the *gdpP* mutant and the LTA-negative suppressor strain with a mutation in *gdpP*, which showed the expected increase in c-di-AMP levels, the 7 suppressor strains chosen for further analysis did not show an increase in cellular c-di-AMP concentrations (Fig. 1C).

### Identification of genomic alterations in the *S. aureus* suppressor strains able to grow in the absence of LTA.

Next, the genomic sequences of the 7 suppressor strains were determined and compared to that of the original *ltaS* mutant strains by using a whole-genome sequencing approach. Mutations were found in *sgtB* (SAUSA300_1855), coding for the monofunctional glycosyltransferase SgtB, and mutations in this gene arose independently in 3 suppressor strains (Table 1). Another strain had a mutation in SAUSA300_1254, coding for a hypothetical membrane protein, and SAUSA300_RS11150, encoding MazE, the antitoxin component of a type II toxin-antitoxin module (Table 1). In the original study by Corrigan et al. (34), mutations found in SAUSA300_1254 were proposed to be accessory and required to further improve the growth of the *gdpP* mutant suppressor strains. Consistent with a previous report (2), a large deletion in *clpX* (SAUSA300_1621), encoding a protein forming part of an ATP-dependent protease, was observed in one strain (Table 1). Finally, a mutation in *vraT* (SAUSA300_1867), coding for the membrane protein VraT and forming part of the VraRST three-component system, was identified. Using our standard genome sequence analysis workflow, an unusually large number (>300) of zero-coverage regions were obtained for suppressor strain S6, preventing us from matching a single-nucleotide polymorphism to the observed growth rescue with high confidence. Therefore, suppressor strain S6 is not listed in Table 1 and was not analyzed further. The mutations identified in the other suppressor strains were subsequently confirmed by fluorescent automated resequencing of the respective genomic region. Some of the observed mutations in *sgtB*, as well as the mutations in *vraT*, *clpX*, and *mazE*, resulted in frameshift mutations and introduction of premature stop codons, suggesting that the absence of the encoded proteins compensates for the lack of LTA.

**TABLE 1:** Genomic variations detected in the suppressor strains, compared the original *ltaS* mutant strains

Strain and reference position(s)^*a*^^,^^*b*^	Type of mutation^*c*^	Reference^*d*^	Allele^*e*^	Frequency (%)^*f*^	Avg quality score^*g*^	Annotations	Amino acid change^*h*^
ANG3711 (S1-*sgtB*)							
2018191	SVN	T	A	100	38.5	SAUSA300_1855; monofunctional glycosyltransferase SgtB	Asn11Tyr
2017952–2018187	DEL					SAUSA300_1855; monofunctional glycosyltransferase SgtB	
ANG3712 (S2-*mazE*)							
1380637	INS		G	100	38.5	SAUSA300_1254; hypothetical membrane protein	Ile291FS
1380639	SNV	C	A	100	38.7		Ser292Tyr
1380641	REP	C	AG	100	38.4		Pro293fs
2188437–2188446	DEL	TATCGGAAAA				SAUSA300_RS11150; antitoxin component of type II toxin-antitoxin module *mazEF*	
ANG3713 (S3-*clpX*)							
1775491	SNV	T	A	100	38.8	SAUSA300_1621; ATP-dependent protease ATP-binding subunit ClpX	Glu148Val
1775495	SNV	T	C	100	38.9		Thr147Ala
1775668	SNV	A	C	100	38.2		Val89Gly
1775500–1775663	DEL						
ANG3714 (S4-*sgtB*): 2017704	SNV	A	C	100	38	SAUSA300_1855; monofunctional glycosyltransferase SgtB	Leu173*
ANG3717 (S7-*vraT*): 2027736	SNV	A	C	100	38.8	SAUSA300_1867; membrane protein Yvfq/VraT	Leu71*
ANG3718 (S8-*sgtB*): 2018107	INS		T	92.6	38.6	SAUSA300_1855; monofunctional glycosyltransferase SgtB	Ser39fs

a Suppressor strains S1, S2, S7, and S8 were derived from strain LAC*Δ*ltaS_S_* (ANG2135), and suppressor strains S3, S4, and S6 were derived from strain LAC*Δ*ltaS_N_* (ANG2134).

b Reference positions indicate the base numbers in the LAC* reference genome (USA300_FPR3757; GenBank accession number CP000255.1).

c DEL, deletion; SNV, single-nucleotide variation; INS, insertion; REP, replacement.

d Base in the LAC* reference genome.

e Base in the sequenced strain.

f Frequency of the specific variant found in the sample reads.

g Average base quality score at the indicated position.

h Amino acid change in the encoded protein in LAC* versus the sequenced strain (fs, frameshift; *, premature stop codon).

### Phenotypic characterization of the LTA-negative *S. aureus* suppressor strains.

**FIG 2:**
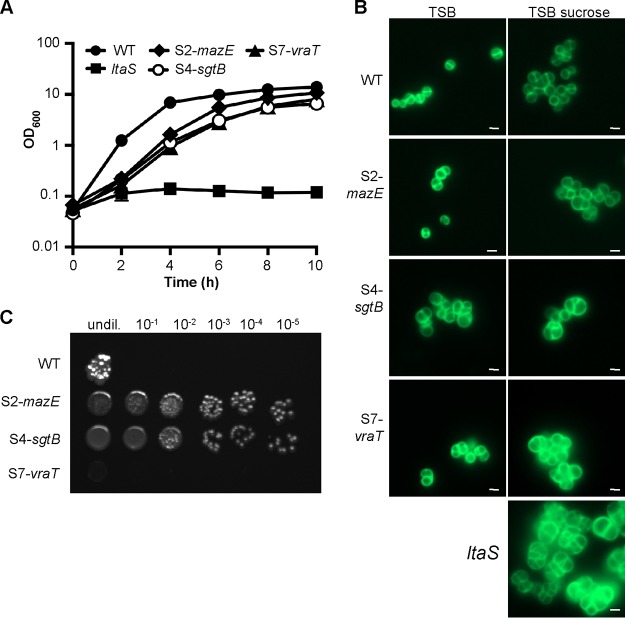
Growth and cell morphology of WT and mutant S. aureus strains. (A) Bacterial growth curves. LAC* (WT strain) and the suppressor strains S2-*mazE*, S4-*sgtB*, and S7-*vraT* were grown overnight in TSB, and the original *ltaS* mutant strain (ANG2135) was grown in TSB with 40% sucrose. The next day, bacterial cells were washed and diluted in TSB to an OD_600_ of 0.05, and the bacterial growth was subsequently monitored over a period of 10 h. The average OD_600_ readings from three experiments were plotted. (B) Microscopic analysis. Bacterial cells from overnight cultures of the same strains as used in panel A were washed, back-diluted, and grown to mid-log phase in TSB or TSB with 40% sucrose, as indicated. The bacterial cells were subsequently stained with BODIPY-vancomycin and analyzed by fluorescence microscopy. (C) Analysis of bacterial growth on Congo red-containing TSA plates. Overnight cultures of the WT strain and the indicated suppressor strains were serially diluted and spotted on TSA plates supplemented with 0.1% Congo red, and the plates were incubated for 48 h at 37˚C. For panels B and C, representative results of three independent experiments are shown.

**FIG 3:**
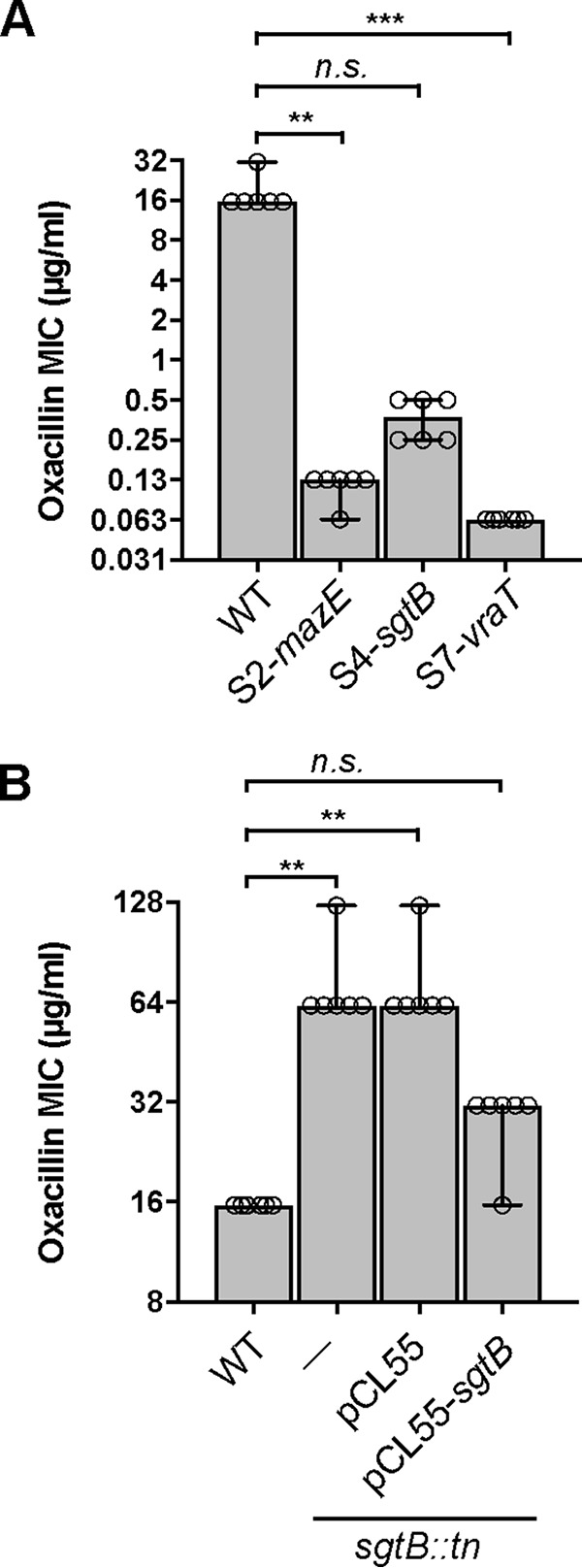
Oxacillin MICs for WT and mutant S. aureus strains. Oxacillin MICs for LAC* (WT strain) and the suppressor strains S2-*mazE*, S4-*sgtB*, and S7-*vraT* (A) and for LAC* (WT strain) and the LAC* *sgtB*::*tn* mutant without an integrative plasmid (−), with an integrative plasmid (pCL55), or with an integrative complementation plasmid (pCL55-*sgtB*) (B) are shown. Experiments were conducted with six biological replicates. Medians and 95% confidence intervals of the oxacillin MIC for all strains are plotted. A Kruskal-Wallis one-way analysis of variance test was performed, which indicated significant differences. Subsequently, Dunn’s tests were performed, and the *P* values were corrected for the multiple comparisons against the WT strain. **, *P* < 0.01; ***, *P* < 0.001; n.s., not significant.

In a previous study, it was shown that S. aureus cells can grow without LTA in the absence of ClpX (2); therefore, we did not further characterize the *clpX* mutant suppressor strain obtained as part of this study. Instead, our analysis focused on suppressor strains with mutations in novel genes, i.e., strains with mutations in *mazE*, *sgtB*, or *vraT*. MazE, the antitoxin component of a type II toxin-antitoxin module and whose is part of the *sigB* regulon, has been shown to be essential for full activity of the alternative sigma factor SigB (37). SigB and members of its regulon enable bacteria to respond rapidly to environmental and antibiotic stresses and also play a role in cell envelope homeostasis (38, 39). Various studies have investigated the role of the VraTSR three-component regulatory system, and this system has been reported to be involved in the induction of the cell wall stressosome, mainly in the presence of cell wall-targeting antibiotics (40–42). Interestingly, the monofunctional glycosyltransferase SgtB, which was also identified as part of our screen, is one of the proteins belonging to the cell wall stressosome whose expression is regulated by VraTSR independent of the presence of cell wall-targeting antibiotics (40). To further characterize the suppressor strains, we first confirmed the growth improvement of strains S2-*mazE*, S4-*sgtB*, and S7-*vraT* when they were propagated in TSB. All 3 suppressor strains grew better than the original *ltaS* mutant strain, and their growth rate was only slightly reduced, compared to that of the wild-type (WT) LAC* strain (Fig. 2A). Next, the cell morphology of the WT strain, the original *ltaS* mutant, and the 3 suppressor strains was assessed by microscopy following staining of the peptidoglycan with fluorescently labeled vancomycin. The cell morphology of the suppressor strains was considerably improved; in particular, for suppressor strain S2-*mazE*, the division site was correctly placed in most cells (Fig. 2B). Next, the susceptibility of the suppressor strains to a number of cell wall-targeting antibiotics was determined. Although the growth and cell morphology of the suppressor strains were improved, the strains remained hypersensitive to the β-lactam antibiotic oxacillin, with MICs reduced ≥32-fold for the different suppressor strains, compared to the WT LAC* strain (Fig. 3A). The susceptibility to the cell wall- or membrane-targeting antibiotics lysostaphin, nisin, vancomycin, and daptomycin was also tested, but no drastic differences were observed (Table 2). Finally, the growth of the 3 different suppressor strains was assessed on plates containing Congo red. Congo red is an anionic azo dye traditionally used for the detection of biofilms in *Staphylococcus*. At higher concentrations, however, it inhibits the growth of S. aureus, and it has been used in the past to indicate differences in the cell wall integrity of different S. aureus strains (43). Recently, the target of Congo red was established as the LTA synthase enzyme LtaS (44). Therefore, our suppressor strains, which have *ltaS* deletions and are able to grow in the absence of LTA, should be resistant to this dye. To test the susceptibility of the suppressor strains, serial dilutions of overnight cultures were spotted on TSA plates containing 0.1% Congo red (Fig. 2C). The suppressor strains carrying mutations in *mazE* and *sgtB* were significantly more resistant to Congo red than was the WT strain, indicating that inactivation of either one of these genes is indeed sufficient to bypass the LTA requirement. However, the suppressor strain with a mutation in *vraT* grew poorly on the Congo red plates, suggesting that this strain might not be a true suppressor strain.

**TABLE 2:** MIC values for different antibiotics

Strain	MIC (µg/ml)^*a*^ of:			
	Lysostaphin	Nisin	Vancomycin	Daptomycin
LAC*	0.125 (0.125, 0.125, 0.125)	16 (16, 16, 16)	4 (4, 4, 4)	16 (16, 16, 16)
S2-*mazE*	0.125 (0.125, 0.125, 0.125)	2–4 (2, 2, 4)	2 (2, 2, 2)	4–8 (8, 8, 4)
S4-*sgtB*	0.125–0.250 (0.125, 0.125, 0.25)	4 (4, 4, 4)	1 (1, 1, 1)	8–16 (16, 8, 8)
S7-*vraT*	0.500 (0.5, 0.5, 0.5)	2–4 (2, 2, 4)	4–8 (8, 4, 4)	8 (8, 8, 8)
LAC*sgtB::*tn*	0.125–0.250 (0.25, 0.125, 0.125)	16–32 (32, 16, 32)	4 (4, 4, 4)	16 (16, 16, 16)
LAC*sgtB::*tn*/pCL55	0.125–0.250 (0.125, 0.125, 0.25)	8–16 (16, 8, 16)	4 (4, 4, 4)	16 (16, 16, 16)
LAC*sgtB::*tn*/pCL55-*sgtB*	0.125 (0.125, 0.125, 0.125)	8–16 (8, 16, 16)	4–8 (4, 8, 4)	16 (16, 16, 16)

a The MIC was defined as the antibiotic concentration that led to >90% growth inhibition, compared to growth without antibiotic. MICs were determined in triplicate. The MIC ranges obtained are shown first, and individual MIC values of the replicate samples are shown in parentheses.

### Introduction of SgtB or MazE in the respective suppressor strain results in the expected growth arrest.

**FIG 4:**
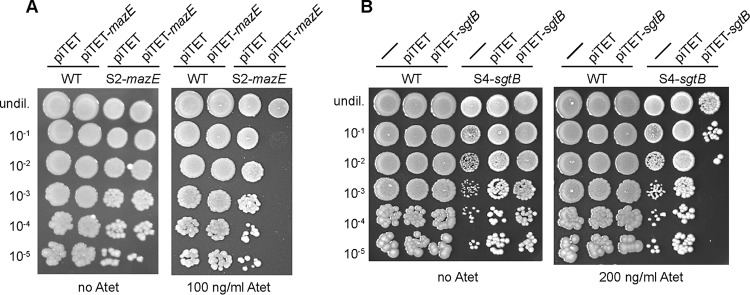
Growth complementation analysis using TSA plates with spot dilutions. (A) S. aureus strains LAC*/piTET, LAC*/piTET-*mazE*, S2-*mazE*/piTET, and S2-*mazE*/piTET-*mazE* were grown overnight in TSB supplemented with chloramphenicol and were washed twice with PBS, and serial dilutions were spotted on TSA plates supplemented with 7.5 μg/ml chloramphenicol, without or with 100 ng/ml Atet. (B) S. aureus strains LAC* (WT strain), LAC*/piTET, LAC*/piTET-*sgtB*, S4-*sgtB*, S4-*sgtB*/piTET, and S4-*sgtB*/piTET-*sgtB* were grown and samples were prepared as described for panel A but without chloramphenicol selection, and dilutions were spotted on TSA plates not supplemented or supplemented with 200 ng/ml Atet (right). Representative plate images from three independent experiments are shown.

**FIG 5:**
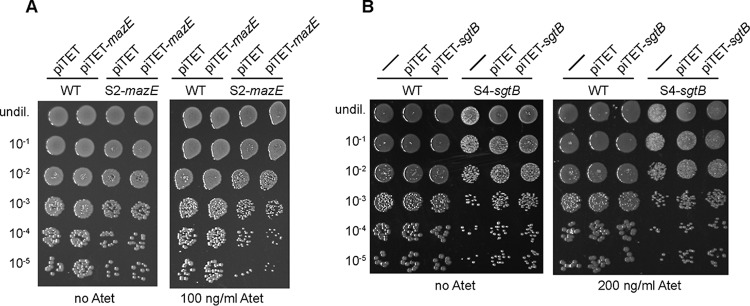
Growth complementation analysis using TSA-sucrose plates with spot dilutions. (A) S. aureus strains LAC*/piTET, LAC*/piTET-*mazE*, S2-*mazE*/piTET, and S2-*mazE*/piTET-*mazE* were grown overnight in TSB supplemented with chloramphenicol and were washed twice with PBS, and serial dilutions were spotted on TSA plates with 40% sucrose supplemented with 7.5 μg/ml chloramphenicol, without or with 100 ng/ml Atet. (B) S. aureus strains LAC* (WT strain), LAC*/piTET, LAC*/piTET-*sgtB*, S4-*sgtB*, S4-*sgtB*/piTET, and S4-*sgtB*/piTET-*sgtB* were grown and samples were prepared as described for panel A but without chloramphenicol selection, and dilutions were spotted on TSA plates with 40% sucrose that were not supplemented or supplemented with 200 ng/ml Atet. Representative plate images from three independent experiments are shown.

The results of the whole-genome sequencing analysis suggested that inactivation of MazE or SgtB was sufficient to allow S. aureus to grow in the absence of LTA. Introduction of a WT copy of *mazE* or *sgtB* into the respective suppressor strain should restore this phenotype and be lethal for the suppressor strains when they are grown in unsupplemented medium but should not have an effect when the bacteria are propagated in medium supplemented with 40% sucrose. In order to test this, plasmids piTET-*mazE* and piTET-*sgtB*, allowing for anhydrotetracycline (Atet)-inducible gene expression, were introduced into the respective suppressor strains. As controls, these plasmids were also introduced into the WT LAC* strain. Serial dilutions of these different strains were spotted onto TSA plates containing 100 ng/ml or 200 ng/ml Atet, for *mazE* or *sgtB* expression, respectively. As expected for successful complementation, the expression of *mazE* prevented the growth of the suppressor strain S2-*mazE* (Fig. 4A) and the expression of *sgtB* prevented the growth of the suppressor strain S4-*sgtB* (Fig. 4B) on TSA but they had no effect when the bacteria were spotted on medium supplemented with 40% sucrose (Fig. 5). Of note, the *mazE* suppressor strain S2-*mazE* contained an additional mutation in a gene coding for a membrane protein of unknown function with locus tag SAUSA300_1254. Introduction of a WT copy of this gene into the suppressor strain did not prevent the growth of this suppressor strain (data not shown). Taken together, the results of this complementation analysis support the notion that inactivation of SgtB or MazE is sufficient to allow S. aureus to grow in the absence of LTA.

### Growth characterization and antibiotic resistance of an *S. aureus* LAC* *sgtB* mutant.

**FIG 6:**
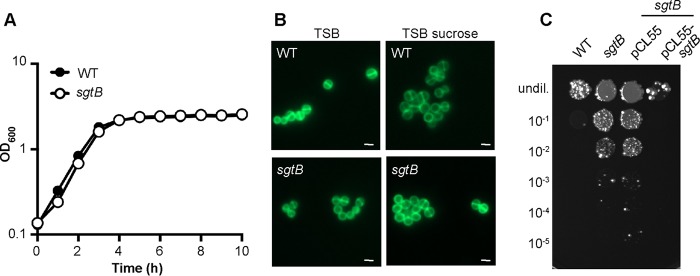
Growth and cell morphology of S. aureus strain LAC*sgtB::*tn*. (A) Bacterial growth curves. Overnight cultures of LAC* (WT strain) and strain LAC*sgtB::*tn* (*sgtB*) were diluted in TSB to an OD_600_ of 0.05, and growth was monitored for 10 h using a plate reader. The average OD_600_ values and SDs from three independent experiments are plotted. (B) Microscopic analysis. S. aureus strains LAC* (WT strain) and LAC*sgtB::*tn* (*sgtB*) were grown overnight in TSB or TSB supplemented with 40% sucrose, stained with BODIPY-vancomycin, and viewed under a fluorescence microscope. (C) Susceptibility to Congo red. Overnight cultures of LAC* (WT strain), the *sgtB* mutant strains LAC*sgtB::*tn* and LAC*sgtB::*tn*/pCL55, and the complementation strain LAC*sgtB::*tn*/pCL55-*sgtB* were serially diluted, and aliquots were spotted on TSA plates supplemented with 0.1% Congo red. For panels B and C, representative results from three independent experiments are shown.

Inactivation of MazE likely has pleotropic effects due to its involvement in the activity of the alternative sigma factor SigB, and the reason why its inactivation allows S. aureus to grow in the absence of LTA could be indirect. Therefore, we next focused on trying to gain a better understanding of the cellular function of the monofunctional peptidoglycan glycosyltransferase SgtB and how its inactivation allows S. aureus to survive in the absence of LTA. To this end, the *sgtB* mutant strain LAC*sgtB::*tn* was constructed by moving the genomic region with a transposon insertion in *sgtB* from the Nebraska transposon mutant library strain NE596 (45) into the LAC* background. SgtB is one of two monofunctional peptidoglycan glycosyltransferases encoded in the S. aureus genome, and the protein can polymerize peptidoglycan chains *in vitro* (46). Although it is dispensable for the growth of S. aureus, SgtB becomes necessary for bacterial survival in the absence of the main glycosyltransferase PBP2 (12, 15, 47). Consistent with these previous observations, no differences in the cell growth and morphology of the *sgtB* mutant S. aureus strain LAC*sgtB::*tn*, compared to the WT LAC* strain, were observed (Fig. 6A and B). Our results indicate that inactivation of SgtB allows S. aureus to survive in the absence of LTA; therefore, the *sgtB* mutant strain should no longer be sensitive to the Congo red dye. To test this, serial dilutions of the WT strain, the *sgtB* mutant, and a complementation strain were spotted on TSA plates containing 0.1% Congo red, which inhibits the LtaS enzyme. Indeed, the *sgtB* mutant strain was considerably more resistant to Congo red than was the WT strain, and this phenotype could be complemented by introducing a functional copy of *sgtB* into the mutant strain (Fig. 6C). Next, we also tested the susceptibility of the *sgtB* mutant and the complementation strain to the cell wall-active antibiotics oxacillin, lysostaphin, nisin, vancomycin, and daptomycin. While no differences in the susceptibility of the *sgtB* mutant to most antibiotics, compared to the WT strain, were observed, a slight (4-fold) and statistically significant increase in resistance to oxacillin was observed (Fig. 3B and Table 2). Taken together, these results indicate that, while no growth or clearly visible morphological differences between the WT strain and the *sgtB* mutant strain were observed, deletion of *sgtB* led to increased Congo red resistance and 4-fold increased oxacillin resistance in our strain background.

### Inactivation of SgtB leading to an increase in peptidoglycan cross-linking in an LTA-negative *S. aureus* strain.

**FIG 7:**
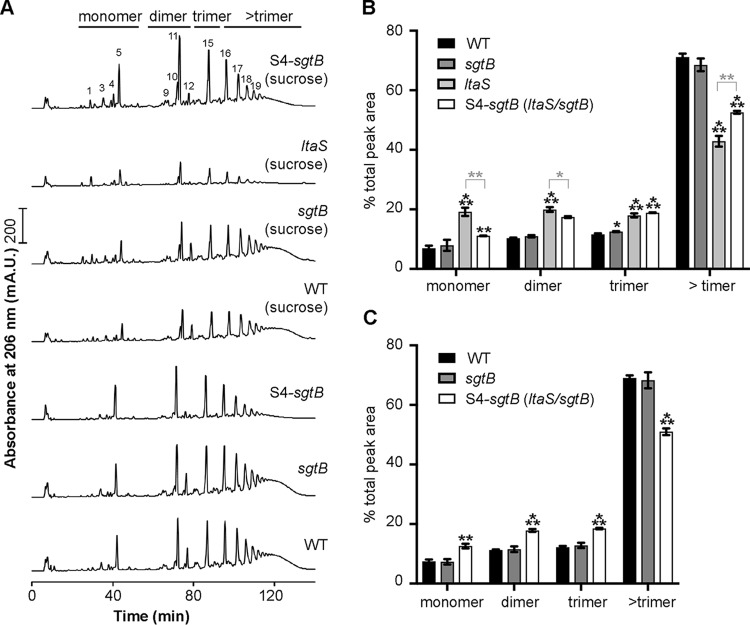
Peptidoglycan analysis of WT and mutant S. aureus strains. (A) HPLC profiles of mutanolysin-digested peptidoglycan samples. Peptidoglycan was isolated and digested with mutanolysin (as described in Materials and Methods) from the S. aureus LAC* strain (WT strain), the LAC*sgtB::*tn* strain (*sgtB*), and the LTA-negative suppressor strain S4-*sgtB* following growth in TSB or from the S. aureus LAC* strain (WT strain), the LAC*sgtB::*tn* strain (*sgtB*), and the LTA-negative suppressor strain S4-*sgtB*, as well as the original *ltaS* mutant strain (ANG2135), following growth in TSB with 40% sucrose. Monomeric, dimeric, trimeric, and greater than trimeric peptidoglycan fragments are indicated above the graphs, and representative profiles from three independent samples are shown. The individual muropeptide peaks were labeled, where possible, with the numbers described by de Jonge et al. (48). (B and C) Quantification of the different peptidoglycan peaks. The peaks corresponding to monomeric, dimeric, trimeric, and greater than trimeric peptidoglycan fragments were integrated and quantified for the strains used in panel A. The combined peak area was set to 100% for each strain, and the average values and SDs from the three independent peptidoglycan isolations were plotted for the strains grown in TSB with sucrose (B) and for the strains grown in TSB (C). Unpaired two-tailed Student’s *t* tests were used to determine statistically significant differences in monomer, dimer, trimer, or greater than trimer fractions between WT and mutant strains. The *P* values obtained were multiplied by Bonferroni correction factors of 3 (B) and 2 (C) because the WT peaks were compared to 3 or 2 different strains, respectively. Statistically significant differences are indicated by black asterisks. For panel B, unpaired two-tailed Student’s *t* tests were also used to determine statistically significant differences in monomer, dimer, trimer, or greater than trimer fractions between the *ltaS* mutant and the other three strains, and statistically significant differences between the *ltaS* mutant and the S4-*ltaS* suppressor strain are shown with gray asterisks. *, *P* < 0.05; **, *P* < 0.01; ***, *P* < 0.001.

Since SgtB is involved in peptidoglycan synthesis and the *sgtB* mutant displayed slightly increased oxacillin resistance, we hypothesized that *sgtB* deletion could somehow “strengthen” the cell wall through alterations in the peptidoglycan structure. In order to investigate this, the muropeptide profiles of peptidoglycan isolated from the WT LAC* strain, the *sgtB* mutant strain LAC*sgtB::*tn*, and the *ltaS sgtB* mutant suppressor strain S4-*sgtB* were determined following the growth of these strains in TSB. In addition, the muropeptide profiles for peptidoglycan isolated from these three strains and from the original *ltaS* mutant strain were determined following growth in TSB supplemented with 40% sucrose (Fig. 7). The WT and *sgtB* mutant strains showed very similar and typical S. aureus muropeptide profiles (Fig. 7A). The chemical structures of several muropeptide fragments in a number of these peaks were determined previously in the seminal paper by de Jonge et al. (48). Where possible, we numbered the peaks as described by de Jonge et al. (48), with peaks 1 to 5 being monomeric, peaks 9 to 14 dimeric, peak 15 trimeric, and peaks 16 and above higher oligomeric muropeptide fragments. Quantification of the monomeric, dimeric, trimeric, and higher oligomeric peptidoglycan fragments showed that the peptidoglycan was highly cross-linked in both strains, with approximately 70% of the UV absorbing material being found in the higher oligomeric fraction (Fig. 7B and C). No clear differences in the muropeptide profiles of the WT strain and the *sgtB* mutant strain, grown either in TSB or in TSB supplemented with 40% sucrose, were found (Fig. 7). In contrast to the WT strain and the *sgtB* mutant strain, a decrease in the higher oligomeric peptidoglycan material was observed for the *ltaS* suppressor strain S4-*sgtB* in both TSB and TSB with sucrose (Fig. 7). Visual inspection of the chromatograms also indicated that peak 12, corresponding to a currently unknown muropeptide, was reduced in S. aureus strains unable to produce LTA, i.e., the original *ltaS* mutant strain and the *S4-ltaS* suppressor strain (Fig. 7A). Perhaps most notably, comparison of the muropeptide profiles of the peptidoglycan isolated from the original *ltaS* mutant strain and the *ltaS sgtB* suppressor strain S4-*sgtB* after growth in TSB with sucrose showed that, while the amount of cross-linked peptidoglycan was reduced in both strains, compared to the WT strain, the peptidoglycan in the suppressor strain was more cross-linked than that in the original *ltaS* mutant strain (Fig. 7A and B). Taken together, our data indicate that deletion of *ltaS* leads to a significant reduction in the amount of cross-linked peptidoglycan in S. aureus. Inactivation of SgtB in a WT strain does not significantly affect the amount of cross-linked peptidoglycan, as might be expected based on the observed increase in oxacillin resistance. In an LTA-negative background strain, however, inactivation of SgtB leads to an increase in peptidoglycan cross-linking, which might explain why an LTA-negative strain can grow in the absence of this monofunctional peptidoglycan glycosyltransferase.

## DISCUSSION

The anionic LTA polymer is a core component of the cell wall that is essential for survival, and a number of studies have shown its importance in various cell processes (for a review, see reference 22). Phenotypes caused by the depletion of LTA in S. aureus involve misplacement and incomplete formation of cell division septa and enlargement of the cells, together ultimately leading to cell lysis (29, 34). However, how LTA mediates these roles is still unknown. In previous work, it was shown that LtaS and other core LTA synthesis proteins physically interact with early- and late-stage cell division proteins, as well as with a number of peptidoglycan synthesis proteins (49). These findings indicate that LTA synthesis enzymes might at least transiently be part of multienzyme complexes, which might help to coordinate LTA synthesis with peptidoglycan synthesis and cell division (49).

We showed previously that S. aureus mutants with increased intracellular c-di-AMP levels could survive without LTA (34). It is now thought that, at high c-di-AMP levels and through the c-di-AMP-dependent regulation of potassium and osmolyte transporters, the internal turgor pressure in the cell might be reduced, so that the compromised LTA-depleted cell wall can sustain the internal pressure (34, 50–52). Indeed, as part of the current study, we showed that the absence of LTA led to a sizable reduction in the amount of cross-linked peptidoglycan in S. aureus (Fig. 7). This finding is consistent with the idea that, in the absence of LTA, the cell wall is likely less able to sustain the high internal turgor pressure, which might also contribute to the increased β-lactam sensitivity of LTA-negative strains observed in this study and previous studies (Table 2) (2, 34). The reason for the reduced amount of peptidoglycan cross-linking is currently not clear but could be due to mislocalization of PBPs in the absence of LTA or PBPs having reduced enzyme activity since the proper ion homeostasis cannot be maintained within the cell wall in the absence of LTA.

The aim of this study was to further elucidate the role of LTA in cell wall assembly and potentially to identify additional proteins involved in the maintenance of cell wall integrity. A suppressor screen followed by whole-genome sequencing revealed mutations in genes coding for ClpX, SgtB, MazE, and VraT (Table 1) that could bypass the requirement for LTA. Further experimentation indicated that the strain with the mutation in *vraT* might not be an actual suppressor strain. In contrast, complementation analysis and growth assays on agar plates containing the azo dye Congo red, which prevents the growth of S. aureus by inhibiting the LtaS enzyme (44), confirmed that inactivating mutations in *sgtB* and *mazE* could bypass the requirement for LTA. In a previous study, it was found that an S. aureus *clpX* mutant readily acquired inactivating mutations in *ltaS*, resulting in the generation of LTA-negative strains (2). Consistent with the previous work, in this study we found a large deletion in *clpX* in one of our LTA-negative suppressor strains (Table 1). ClpX is a protein-folding chaperon, which recognizes and targets proteins for degradation to the ClpP protease component. In the absence of ClpX, S. aureus cells become smaller, show increased production of autolysins, and have a severe growth defect at temperatures of 30˚C or lower (2). The introduction of loss-of-function mutations in the gene coding for the LTA synthase LtaS in a *clpX* mutant alleviates some of these effects; this is perhaps due to LTA depletion having the opposite effect, leading to increased cell size and decreased autolysis, as reported in some previous publications (2, 31).

Mutations in the monofunctional glycosyltransferase SgtB were the most prevalent mutations that arose in our suppressor screen. Previous studies showed that SgtB is not essential for cell survival and, as reported in this study and a previous study, an *sgtB* mutant strain does not show any obvious growth or morphological differences, compared to a WT strain, under standard growth conditions (Fig. 6) (15). Interestingly, we found that the *sgtB* mutant strain LAC*sgtB::*tn* was 4-fold more resistant to the cell wall-targeting antibiotic oxacillin and this phenotype could be complemented by introduction of a WT copy of *sgtB* (Table 2). These results are in accordance with previous reports, in which strains with mutations in genes that compensate for the lack of LTA, such as *gdpP* and *clpX* mutant strains, also show increased oxacillin resistance (2, 34).

The increased resistance of the *sgtB* mutant to oxacillin prompted us to investigate the peptidoglycan structure of an *sgtB* mutant in more detail, as we hypothesized that, in the absence of SgtB, changes such as increased cross-linking might be observed, which could potentially explain the increased resistance. We could not detect any obvious changes in the muropeptide profile of an *sgtB* mutant strain, compared to a WT strain (Fig. 7). However, the original *ltaS* mutant strain showed a significant reduction (around 30%) in the higher oligomeric cross-linked peptidoglycan (Fig. 7). Perhaps most importantly, the peptidoglycan isolated from the *sgtB ltaS* double mutant S4-*sgtB* suppressor strain showed an increase in peptidoglycan cross-linking, compared to the original *ltaS* mutant strain (Fig. 7). We speculate that this increase in peptidoglycan cross-linking could potentially strengthen the cell wall to better sustain the high internal turgor pressure and might be at least partly responsible for the observed growth improvement.

Bacterial two-hybrid studies have indicated that the S. aureus SgtB protein interacts with SgtA, PBP1, PBP2, and PBP2A (15). Therefore, SgtB and the main glycosyltransferase PBP2 might compete for substrate during peptidoglycan biosynthesis, and inactivation of the former might increase the substrate availability and activity of the latter. This might then aid in the delivery of substrate to the transpeptidase domain of the bifunctional PBP2 enzyme, resulting in increased cross-linking. Alternatively, SgtB could also affect the function of PBP4, which has been shown to be responsible for hyper-cross-linking of the staphylococcal peptidoglycan (53). Clearly, more experiments are needed to clarify the complex nature of peptidoglycan biosynthesis in S. aureus and the changes observed in the absence of LTA.

Mutations in MazE could potentially also be involved in changes in the cell wall structure of S. aureus. The MazEF type II toxin-antitoxin system is part of the *sigB* regulon and has been shown to be required for full activity of the alternative sigma factor SigB (37). It has also been reported that overexpression of σ^B^ causes cell wall thickening in S. aureus and increased resistance to cell wall-targeting antibiotics (38). Therefore, inactivation of MazE, as observed in one of our suppressor strains, could potentially also affect cell wall homeostasis via its effect on SigB.

In summary, our results suggest that, in the absence of LTA, peptidoglycan in S. aureus becomes less cross-linked, which might weaken the cell wall and cause it to become less able to sustain the high internal turgor pressure, ultimately leading to cell lysis. The suppressor mutations in *sgtB* (and perhaps also in some of the other genes observed in our screen) help the cell survive this detrimental effect by altering and strengthening the cell wall, likely allowing the cell wall to better withstand the high internal turgor pressure.

## MATERIALS AND METHODS

### Bacterial strains and culture conditions.

All bacterial strains used in this study are listed in Table 3. Escherichia coli strains were cultured in lysogeny broth (LB) and Staphylococcus aureus strains were cultured in TSB at 37°C with aeration, unless otherwise stated. When appropriate, the growth medium was supplemented with antibiotics and inducers as follow: for E. coli cultures, 100 μg/ml ampicillin, 10 μg/ml chloramphenicol, and 30 μg/ml kanamycin; for S. aureus cultures, 10 μg/ml chloramphenicol for plasmid selection or 7.5 μg/ml chloramphenicol for chromosomally integrated plasmid selection, 10 μg/ml erythromycin, and 90 μg/ml kanamycin. The inducer Atet was used at a concentration of 100 or 200 ng/ml in agar plates and 50 ng/ml in broth.

**TABLE 3:** Bacterial strains used in this study

Species and strain	Description^*a*^	Reference or source
Escherichia coli		
XL1-Blue	Cloning strain; Tet^r^; ANG127	Stratagene
IM08B	E. coli-S. aureus shuttle strain; ANG3723	56
ANG3732	IM08B/pCL55 Amp^r^	52
ANG3926	XL1-Blue/piTET-*sgtB* Amp^r^	This study
ANG3927	IM08B/piTET-*sgtB* Amp^r^	This study
ANG3928	IM08B/piTET Amp^r^	57
ANG4708	XL1-Blue/pCL55-*sgtB* Amp^r^	This study
ANG4707	IM08B/pCL55-*sgtB* Amp^r^	This study
ANG4727	XL1-Blue/piTET-*mazE* Amp^r^	This study
ANG4728	IM08B/piTET-*mazE* Amp^r^	This study
Staphylococcus aureus		
LAC*	Erm-sensitive community-acquired MRSA LAC strain; ANG1575	58
NE596	JE2*sgtB*::*tn*, Nebraska Transposon Mutant Library strain; Erm^r^; ANG3930	45
ANG1961	LAC*gdpP::*kan* Kan^r^	34
ANG2134	LAC*Δ*ltaS_*N*_*::*erm* pCN38, isolated on 7.5% NaCl; Cam^r^ Erm^r^	34
ANG2135	LAC*Δ*ltaS_*S*_*::*erm* pCN38, isolated on 40% sucrose; Cam^r^ Erm^r^	34
ANG2137	LAC*Δ*ltaS_*N*_*::*erm* suppressor S6, original colony; Erm^r^	34
ANG2140	LAC*Δ*ltaS_*S*_*::*erm* suppressor S7, original colony; Erm^r^	34
ANG2143	LAC*Δ*ltaS_*S*_*::*erm* suppressor S8, original colony; Erm^r^	34
ANG2434	LAC*Δ*ltaS_*S*_*::*erm* suppressor with *gdpP* mutation, pass 4 (short: US3); Erm^r^	34
ANG3694	LAC*Δ*ltaS_*S*_*::*erm* suppressor S1, original colony; Erm^r^	This study
ANG3698	LAC*Δ*ltaS_*S*_*::*erm* suppressor S2, original colony; Erm^r^	This study
ANG3703	LAC*Δ*ltaS_*S*_*::*erm* suppressor S3, original colony; Erm^r^	This study
ANG3707	LAC*Δ*ltaS_*S*_*::*erm* suppressor S4, original colony; Erm^r^	This study
ANG3711	LAC*Δ*ltaS_*S*_*::*erm* suppressor S1, pass 4 (short: S1-*sgtB*); Erm^r^	This study
ANG3712	LAC*Δ*ltaS_*S*_*::*erm* suppressor S2, pass 4 (short: S2-*mazE*); Erm^r^	This study
ANG3713	LAC*Δ*ltaS_*N*_*::*erm* suppressor S3, pass 4 (short: S3-*clpX*); Erm^r^	This study
ANG3714	LAC*Δ*ltaS_*N*_*::*erm* suppressor S4, pass 4 (short: S4-*sgtB*); Erm^r^	This study
ANG3716	LAC*Δ*ltaS_*N*_*::*erm* suppressor S6, pass 4 (short: S6); Erm^r^	This study
ANG3717	LAC*Δ*ltaS_*S*_*::*erm* suppressor S7, pass 4 (short: S7-*vraT*); Erm^r^	This study
ANG3718	LAC*Δ*ltaS_*S*_*::*erm* suppressor S8, pass 4 (short: S8-*sgtB*); Erm^r^	This study
ANG4054	LAC*/piTET Cam^r^	57
ANG4056	LAC*/piTET-*sgtB* Cam^r^	This study
ANG4057	S4-*sgtB*/piTET Erm^r^ Cam^r^	This study
ANG4058	S4-*sgtB*/piTET-*sgtB* Erm^r^ Cam^r^	This study
ANG4059	LAC*sgtB::*tn* Erm^r^	This study
ANG4710	LAC*sgtB::*tn*/pCL55 Erm^r^ Cam^r^	This study
ANG4711	LAC*sgtB::*tn*/pCL55-sgtB Erm^r^ Cam^r^	This study
ANG4725	S2-*mazE*/piTET Erm^r^ Cam^r^	This study
ANG4729	LAC*/piTET-*mazE* Cam^r^	This study
ANG4730	S2-*mazE*/piTET-*mazE* Erm^r^ Cam^r^	This study

a Tet^r^, tetracycline resistant; Amp^r^, ampicillin resistant; Erm, erythromycin; Erm^r^, erythromycin resistant; Kan^r^, kanamycin resistant; Cam^r^, chloramphenicol resistant.

### Plasmid and strain construction.

Strains and primers used in this study are listed in Tables 3 and 4, respectively. Strain LAC*sgtB::*tn* was generated by transduction using phage Φ85. In this manner, the *sgtB* region containing a transposon insertion in *sgtB* was transduced from the Nebraska transposon mutant library strain NE596 (45) into strain LAC*. Plasmids piTET-*sgtB* and piTET-*mazE* for Atet-inducible expression of *sgtB* and *mazE* in S. aureus were generated by amplifying *sgtB* or *mazE* from LAC* chromosomal DNA using the primer pair ANG2268/ANG2269 or ANG2743/ANG2744, respectively. The PCR products and plasmid piTET were digested with AvrII and SacII, ligated, and then transformed into E. coli XL1-Blue, yielding strains XL1-Blue/piTET-*sgtB* (ANG3926) and XL1-Blue/piTET-*mazE* (ANG4727). The plasmids were shuttled through E. coli strain IM08B (ANG3927 and ANG4728) and subsequently electroporated into LAC*, yielding strains LAC*/piTET-*sgtB* (ANG4056) and LAC*/piTET-*mazE* (ANG4729), or into the respective suppressor strains, yielding strains S4-*sgtB*/piTET-*sgtB* (ANG4058) and S2-*mazE*/piTET-*mazE* (ANG4730). The plasmid pCL55-*sgtB* for complementation and expression of *sgtB* from its native promoter was generated by amplifying the *sgtB* gene, including its native promoter region, from LAC* chromosomal DNA using primers ANG2270 and ANG2271. The PCR product and plasmid pCL55 were digested with EcoRI and BamHI, ligated, and then transformed into E. coli XL1-Blue, yielding strain XL1-Blue/pCL55-*sgtB* (ANG4708), shuttled through IM08B (ANG3923), and subsequently electroporated into the *sgtB* mutant strain, yielding strain LAC*sgtB::*tn*/pCL55-*sgtB* (ANG4711). WT LAC* and mutant S. aureus strains with the empty vectors piTET and pCL55 (Table 3) were used as control strains in several experiments. The sequences of all plasmid inserts were verified by automated fluorescence sequencing at GATC Biotechnology.

**TABLE 4:** Primers used in this study

Primer identification	Primer name	Sequence^*a*^
ANG2229	sausa300_1867 Fw seq	GAAGCATTTCAAATTCAATGTGCAAG
ANG2230	sausa300_1867 Rv seq	GAAGCATTTCAAATTCAATGTGCAAG
ANG2231	sgtB Fw seq	GTCGTCTCTATTGGCATTTAATAGGG
ANG2232	sgtB Rv seq	AATAATGAAGTACTAATCAAGTGGC
ANG2233	sausa300_1254 Fw seq	CATTTTGAAGAAAGGATTAATCAA
ANG2234	sausa300_1254 Rv seq	AATAATGAAGTACTAATCAAGTGGC
ANG2235	clpX Fw seq	GAAGGAACTAAAGAAGATTAATCTTC
ANG2236	clpX Rv seq	CTGGATATTGTTCTTCTTTTACTGCAC
ANG2268	5-AvrII-sgtB-pCL55iTet	CATG CCTAGGTTAAAAGAAGGAGCAAACGCATG
ANG2269	3-SacII-sgtB-pCL55iTet	CATG CCGCGGTTAACGATTTAATTGTGACATAGCC
ANG2409	5- EcoRI-promSgtB-pCL55	CATG GAATTCAAGTATTGTGGTTATCGATTG
ANG2410	3- BamH1-promSgtB-pCL55	CATG GGATCCTTAACGATTTAATTGTGACATAGCC
ANG2740	5-mazE SNP seq	AGATAATCATAGAGAAAGTCCACAGTCG
ANG2741	3-mazE SNP seq	TAAGTACGTCAGTTTTTCTTTCAATCGT
ANG2743	5-AvrII-mazE-pCL55iTet	CATG CCTAGGGGAATCAATTGGAGGTTCTCATATG
ANG2744	3-SacII-mazE-pCl55iTet	CATG CCGCGGTCATTCATTCGTTGAATTAGAAGATAAATATG

a Restriction site sequences are underlined.

### Bacterial growth curves.

S. aureus LAC* and the indicated *ltaS* suppressor strains were grown overnight in TSB containing the relevant antibiotic. The original *ltaS* mutant strains were grown in TSB containing either 7.5% NaCl or 40% sucrose. Overnight cultures were washed three times in TSB and diluted to a starting optical density at 600 nm (OD_600_) of 0.05. Cultures were incubated at 37°C with aeration, and OD_600_ values were determined at 2-h intervals. WT LAC* and the *sgtB* mutant strain LAC*sgtB::*tn* were grown overnight in TSB or TSB supplemented with 10 µg/ml erythromycin. The next day, bacteria were washed three times in TSB and diluted to a starting OD_600_ of 0.05, and 200 μl was placed in a 96-well microtiter plate. Growth was monitored for 10 h using a SPECTROstar Nano plate reader (BMG Labtech). All growth curves were determined in triplicate, with average values and standard deviations (SDs) being plotted.

### Fluorescence microscopy analysis.

Cells from 1 ml of overnight culture were collected by centrifugation and washed three times in phosphate-buffered saline (PBS) (pH 7.4), and 150 μl of the cell suspensions was applied to polylysine (0.1% [wt/vol])-treated coverslips. The coverslips coated with the bacteria were incubated for 20 min with 100 µl of a 1-μg/ml boron-dipyrromethene (BODIPY)-vancomycin (Molecular Probes) solution in water, washed, and mounted on glass slides containing 20 µl Vectashield (Vector Laboratories). Slides were viewed under a Zeiss Axio Imager A2, using a green fluorescent protein (GFP) filter set, and images were captured with an AxioCam MRc Rev.3 camera and analyzed using Zen Pro 2012 SP2 software. The experiment was performed in triplicate.

### MIC determination.

Overnight cultures of the WT strain, the *sgtB* mutant, and the complementation strain, as well as the indicated suppressor strains, were grown overnight in TSB. The next day, cultures were adjusted to an OD_600_ of 0.05 in TSB, and 100-μl aliquots of the suspensions were incubated in 96-well plates with 2-fold dilutions of various antimicrobials, at the following starting concentrations: oxacillin, 500 µg/ml or 1 μg/ml as appropriate; daptomycin, 32 μg/ml; lysostaphin, 2 μg/ml; vancomycin, 32 μg/ml; nisin, 64 μg/ml. Oxacillin- and daptomycin-containing wells were supplemented with 2% (wt/vol) NaCl and 0.23 mM CaCl_2_, respectively. Plates were incubated for 24 h at 37°C, with shaking at 500 rpm. MICs were determined as the concentrations of antibiotic at which growth was inhibited by >90%, compared to growth without the antibiotic.

### LTA detection by Western blotting.

LTA extraction and detection by Western blotting were performed as described previously (54). Briefly, samples were prepared from 1-ml overnight cultures normalized based on OD_600_ readings, i.e., cells from 1-ml cultures with an OD_600_ of 6 were suspended in 90 µl 2× SDS protein sample buffer. Ten-microliter aliquots of these samples were separated on 15% SDS-polyacrylamide gels, and the material was subsequently transferred to polyvinylidene difluoride (PVDF) membranes. LTA was detected using a polyglycerolphosphate-specific LTA monoclonal antibody (Hycult Biotechnology) at a 1:4,000 dilution and a horseradish peroxidase (HRP)-conjugated anti-mouse IgG antibody (Cell Signaling Technologies, USA) at a 1:10,000 dilution. The blots were developed by enhanced chemiluminescence (ECL) using the Clarity Western ECL blotting substrate (Bio-Rad) and were imaged using the ChemiDoc Touch imaging system (Bio-Rad). Western blots were performed in triplicate, and representative results are shown.

### Detection of WTA by Alcian blue/silver staining.

Flasks with 60 ml TSB supplemented with the appropriate antibiotics were inoculated with single colonies of WT LAC* and different suppressor strains, and the cultures were incubated overnight (18 h) at 37°C, with shaking. The original *ltaS* mutant was grown in TSB supplemented with 40% sucrose and was incubated 6 to 8 h longer. Cells from an OD_600_ equivalent of 120 were harvested by centrifugation, and the bacterial pellet was stored at −20°C for further processing. WTA extraction and detection by Alcian blue/silver staining following SDS-PAGE analysis was performed as described by Covas et al. (55). Briefly, cells were washed in 20 ml buffer 1 [50 mM 2-(*N*-morpholino)ethanesulfonic acid (MES) (pH 6.5)], resuspended in 20 ml buffer 2 (buffer 1 with 4% [wt/vol] SDS), and boiled for 60 min. Next, the cells were washed with 20 ml buffer 2 and, after transfer to 2.0-ml reaction tubes, washed once more with 1.6 ml buffer 2, 1.6 ml buffer 3 (buffer 1 with 2% [wt/vol] NaCl), and finally 1.6 ml buffer 1. The samples were suspended in 1.6 ml buffer 4 (20 mM Tris-HCl [pH 8.0], 0.5% [wt/vol] SDS) and incubated for 4 h at 50°C, with shaking, following the addition of 2 µl of proteinase K solution (20 mg/ml) from Tritirachium album. Next, the cells were collected by centrifugation, washed once with 1.6 ml buffer 3, and washed three times with 1.6 ml of water. To release WTA, the pellets were suspended in 1 ml of 0.1 mM NaOH, and the samples were incubated for 16 h at 25°C. Next, the samples were centrifuged, 30 µl of the supernatant (containing WTA) was separated on native Tris-Tricine polyacrylamide gels, and WTA was visualized by Alcian blue/silver staining (55).

### Whole-genome sequencing.

WT LAC*, LAC*Δ*ltaS_N_*, LAC*Δ*ltaS_S_*, and indicated suppressor strains were cultured overnight at 37°C, cells were harvested, and genomic DNA was extracted. Genome sequencing was performed by MicrobesNG (University of Birmingham), using an Illumina platform and a 250-bp paired-end read kit. Sequence analysis was performed using the CLC Genomics Workbench software package. First, the LAC* reads were aligned against the published USA300 FPR3757 genome sequence (RefSeq accession number NC_007793.1) and assembled into a reference contig, and the USA300 FPR3757 annotation was transferred onto the LAC* sequence. Next, the Illumina reads for the original *ltaS* mutant strains LAC*Δ*ltaS_S_* and LAC*Δ*ltaS_N_*, as well as the 7 different suppressor strains, were mapped onto the assembled LAC* sequence, and high-frequency (>65%) and good-quality base changes were identified using the CLC Genomics Workbench software package. Genomic alterations found in the suppressor strains but not present in the original *ltaS* mutant strain are summarized in Table 1.

### Peptidoglycan isolation and analysis.

Overnight cultures of S. aureus strains LAC*, S4-*sgtB*, and LAC*sgtB::*tn* were prepared in TSB or TSB supplemented with 40% sucrose, and cultures of the original *ltaS* mutant strain LAC*Δ*ltaS_N_* (ANG2134) were prepared in TSB supplemented with 40% sucrose. The next day, cells were back-diluted in 2 liters of the same growth medium, to an OD_600_ of 0.05. The cultures were grown at 37°C until they reached an OD_600_ of approximately 1.5 and were cooled on ice, and cells were collected by centrifugation. Peptidoglycan was purified and digested with mutanolysin as described previously (34, 48). High-performance liquid chromatography (HPLC) analysis of the digested peptidoglycan material was performed as described previously (48), and the muropeptide profiles were determined in triplicate for each strain and growth condition. For the quantification of monomeric, dimeric, trimeric, and higher oligomeric peptidoglycan material, the peaks were integrated. The total peak area for each muropetide profile was determined and set to 100%, the percentages of monomeric, dimeric, trimeric, and higher oligomeric peaks were calculated, and the average values and SDs from the three profiles were determined and plotted.

### c-di-AMP quantification by competitive ELISA.

Five-milliliter aliquots of TSB were inoculated with single colonies of S. aureus LAC* (WT strain), strain LAC* *gdpP*::*kan*, strain US3 (LAC*Δ*ltaS_S_*::*erm* suppressor with a mutation in *gdpP*), and the different LTA-negative suppressor strains, and the tubes were incubated for 8 h at 37°C. Next, the cultures were back-diluted in 10 ml TSB to an OD_600_ of 0.05 and were grown for 15 h at 37°C. Bacterial cells from these cultures were collected by centrifugation, cell lysates were prepared, and the cellular c-di-AMP levels were determined using a previously described competitive ELISA method (35, 36). However, instead of determining c-di-AMP levels based on a standard curve, the *A*_490_ values were directly compared. To this end, the *A*_490_ readings obtained for samples derived from the WT strain were set to 100%, and percentage values were calculated for the samples derived from the other strains. Thee independent experiments were performed, with three technical replicates, and the average values and SDs for the percentage values were determined for each strain, compared to the WT strain. Of note, since this was a competitive ELISA, decreases in *A*_490_ readings represented increases in cellular c-di-AMP levels.

### Accession number(s).

The Illumina short read for the WT LAC* was published previously (35) and has been deposited in the European Nucleotide Archive under study accession number PRJEB14759. The Illumina reads for the *ltaS* mutants and the *ltaS* suppressor strains were deposited in the European Nucleotide Archive under study accession number PRJEB29420.
